# Supplementary material for: Identification of Extrachromosomal Linear microDNAs Interacted with microRNAs in the Cell Nuclei
Source: Cells. 2019 Feb 1;8(2):111. doi: 10.3390/cells8020111 (PMC6406244; doi:10.3390/cells8020111)
Supplement: Supplementary file 1 [file cells-08-00111-s001.zip › manuscript-revise/manuscript-revise-track change.docx]

Article

Identification of extrachromosomal linear microDNAs interacted with microRNAs in the cell nuclei

Teng Sun ^1,^ *, Kun Wang ^2^, Cuiyun Liu ^2^, Yin Wang ^2^, Jianxun Wang ^2,^ * and Peifeng Li ^2,^*

^1^ Key Laboratory of Cellular Physiology, Ministry of Education, Department of Physiology, Shanxi Medical University, Taiyuan 030001, China; tengsun@qdu.edu.cn

^2^ Institute for Translational Medicine, Qingdao University, Qingdao, China; wangk696@yeah.net (K.W.); zhuanhuacuiyun@126.com (C.-Y.L.); wangying@sibs.ac.cn (Y.W.)

***** Correspondence: tengsun@qdu.edu.cn (T.S.); wangjx@qdu.edu.cn (J.-X.W.); peifliqdu@126.com (P.-F.L.); Tel.: +86-(351)-4135117

Received: date; Accepted: date; Published: date

**Abstract:** Extrachromosomal DNA exists in two forms: covalently closed circular and linear. While diverse types of circular extrachromosomal DNA have been identified with validated in vivo functions, little is known about linear extrachromosomal DNA. In this study, we identified small, single-stranded linear extrachromosomal DNAs (SSLmicroDNAs) in the nuclei of mouse heart, mouse brains, HEK293, and HeLa cells. We used a pull-down system based on the single-stranded DNA binding protein RecAf. We found that SSLmicroDNAs aligned predominantly to intergenic and intragenic regions of the genome, owned a variety of single nucleotide polymorphism sites, and strongly associated with H3K27Ac marks. The regions were tens to hundreds of nucleotides long, periodically separated by AT, TT, or AA dinucleotides. It has been demonstrated that SSLmicroDNAs in the nuclei of normal cells target microRNAs , which regulate biological processes. In summary, our present work identified a new form of extrachromosomal DNAs, which function inside nuclei and interact with microRNAs. This finding provides a possible research field into the function of extrachromosomal DNA.

**Keywords:** extrachromosomal DNAs; small single-stranded linear extrachromosomal DNAs (SSLmicroDNAs); sequence analysis; microRNAs; cell nucleus

1. Introduction

Extrachromosomal DNA—DNA molecules separated from chromosomes—exist in two main forms: covalently closed circular and linear DNA [[1-5](#_ENREF_1)]. Mitochondrial DNA are located in mitochondria and represent typical extrachromosomal circular DNA (eccDNA). This type of DNA encodes functional genes and contributes to the nuclear genomes’ instability [[6](#_ENREF_6)]. Small polydispersed circular DNA, double minute chromosomes, episomes, minichromosomes, autonomously replicating sequences, telomeric-circles, B- and T-cell receptor excision circles, and extrachromosomal elements induced by c-myc oncogene deregulation and resulting in genomic instability were discovered as extrachromosomal circular DNA molecules with unique properties and relevant in vivo functions, which are produced randomly or non-randomly [[1](#_ENREF_1),[7-10](#_ENREF_7)]. In recent years, an increasing number of eccDNAs has been identified in many organisms. Preliminary research links eccDNAs to cancer [[11](#_ENREF_11),[12](#_ENREF_12)]. Recently, much attention has focused on a class of small circular extrachromosomal DNAs (microDNAs), with short length and unique features. microDNAs have been found ubiquitously expressed in mammalian cells, including mouse brain, heart, kidney, liver, lung, skeletal muscle, sperm, spleen, testis, and thymus as well as cancer cell lines. Several hypotheses exist regarding the origin of microDNAs, including genome microdeletion, mismatch repair pathways, and transcriptional activity [[7](#_ENREF_7),[13](#_ENREF_13)].

Studies have demonstrated wide distribution and function of diversiform eccDNAs in animals, plants, and microorganisms [[1](#_ENREF_1),[7](#_ENREF_7),[8](#_ENREF_8)], while extrachromosomal linear DNAs only exist in certain organisms or at certain states [[2](#_ENREF_2),[4](#_ENREF_4),[14-18](#_ENREF_14)]. Linear plasmid pAM1 was identified outside chromosomes in the Actinoplanes species [[17](#_ENREF_17)]. Small linear extrachromosomal DNAs exist in mitochondria of plant and fungi [[2](#_ENREF_2),[16](#_ENREF_16)]. Creophilus maxillosus and Candida albicans have been shown to contain genome-independent linear ribosomal DNA (rDNA), with functional polarity in [[15](#_ENREF_15)]. Some linear extrachromosomal DNAs appear during pathological states. Twelve linear extrachromosomal plasmids with sequence similarities to plasmids in Borrelia burgdorferi type strain isolate B31 were discovered in isolates of Lymes disease agent B. burgdorferi [[5](#_ENREF_5),[18](#_ENREF_18)]. During retroviral infections, numerous un-integrated retroviral DNAs accumulate outside chromosomes in infected cells [[14](#_ENREF_14)]. It is not yet clear whether new forms of extrachromosomal linear DNA exist in higher organisms, and whether they perform a certain function.

MicroRNAs (miRNAs) are a kind of ~22nt long non-coding RNAs, which play a critical role in regulating gene expression. They usuallytarget the 3’-untranslated region (UTR) or mRNA coding sequences (CDS) to prevent mRNA translation or promote degradation [[19](#_ENREF_19)]. miRNAs are now a fundamental part of the entire regulatory network of biological process, including cell proliferation, differentiation, and death both during physiological and pathological states [[19-22](#_ENREF_19)]. Many miRNAs are found in the nucleus where they regulate multiple process, such as chromatin remodeling [[23](#_ENREF_23)], transcriptional silencing [[24](#_ENREF_24)], mRNA alternative splicing [[25](#_ENREF_25),[26](#_ENREF_26)], and microRNA maturation [[20](#_ENREF_20),[27](#_ENREF_27)].

Our present work identified a new form of extrachromosomal linear DNA, single-stranded linear microDNAs (SSLmicroDNAs), which are found in the nuclei of multiple cell types, including adult mouse hearts, mouse brains, HEK293 cells, and HeLa cells. We analysed the unique features of SSLmicroDNAs, and we proposed several hypotheses. Our results revealed that SSLmicroDNAs interact with microRNAs in nuclei, implying a potential role in microRNA regulatory pathways.

2. Materials and Methods

2.1. Isolation of extrachromosomal single-stranded linear DNAs and related microRNAs

Nuclei were icubated with a weak lysis buffer (20 mM Tris-HCl (pH 7.5), 200 mM NaCl, 2.5 mM MgCl2, 0.05% Igepal, 60 U RNase inhibitor, 1 mM DTT, and proteinase inhibitor) and shaken overnight at 4℃. The lysates were pre-cleared by centrifugation at 3000 rpm for 10 min, followed by incubation with single-stranded DNA binding protein RecAf (New England biolabs@ Inc.), and 2.4 mM ATP at 37 ℃ for 3 h. NTA-Ni agarose beads were incubated with RecAf-DNA complexes at 4 ℃ with shaking for 4 h, then washed with Ni-washing buffer (20 mM Hepes (pH 7.5), 10% glycerol, 0.3 M NaCl, 0.2% Triton X-100, 25 mM imidazole, 10 mM beta-mercaptoethanol, and 0.5 mM PMSF) three times. The beads were divided into two aliquots: one was eluted using Ni-Elution buffer (20 mM Hepes (pH 7.5), 10% glycerol, 0.3 M NaCl, 0.35 M Imedazole, 10 mM beta-mercaptoethanol, and 0.5 mM PMSF) to extract total single-stranded DNA, while the other was used to extract related RNAs using Trizol (InvitrogenTM life technology).

2.2. SSLmicroDNA library construction and sequencing

Total single-stranded linear DNAs were ligated with two specific double-stranded adaptors— adaptor A or adaptor B. Adaptor A forward sequence was: 5’- CACACTCTTTCCCTACACGACGCTCTTCCGATCTTTGCNNNNNN- 3’, and the reverse sequence was 5’- GCAAAGATCGGAAGAGCGTCGTGTAGGGAAAGAGTGTG- 3’. Adaptor B forward sequence was 5’-NNNNNNGTTCAGAGTTCTGCGACAGGAGAGGTCGTATGCCGTCTTCTGCTTG-3’, and the reverse sequence was 5’-CAAGCAGAAGACGGCATACGACCTCTCCTGTCGCAGAACTCTGAAC-3’. The adaptors could only ligate to single-stranded linear DNA through sticky ends and six random bases pairing to single strands. The complementary strand was synthesized and amplified by a PCR reaction with the forward primer 5’-CAAGCAGAAGACGGCATACGA-3’ and the reverse primer 5’-ACACTCTTTCCCTACACGAC-3’. DNA mixtures ranging from less than 500 bp, 500–1000 bp, and 1000–2000 bp were collected and cloned into the pZeroback T vector (TianGen biotech co., LTD). Sequencing was performed using the TSINGKE Biological Technology.

2.3. Atomic force microscopy

DNA was imaged using atomic force microscopy, the experiment was conducted following the protocol described in Reference [[28](#_ENREF_28)]. Briefly, a drop of DNA (5 ng/μl) with 5 mM MgCl2 was placed on the surface of freshly cleaved mica, and left for 2 min at room temperature. The mica was then rinsed with 1 ml water, blotted with filter paper, and dried by the flow of compressed nitrogen for 2 min. Samples in mica were scanned using a Digital Instrument’s MultiMode scanning probe on a Nanoscope IIIa (Veeco) microscope operating in Tapping Mode.

2.4. Fluorescence in situ hybridization

Fluorescence in situ hybridization was performed following after modifying the method described in Reference [[29](#_ENREF_29)]. Briefly, cells were grown on glass coverslips, coated with poly-L-lysine (Sigma-Aldrich), and fixed with 4% paraformaldehyde for 30 min at room temperature. They were treated consecutively with 4.5% sucrose buffer for 1 h 4 times, fresh 0.1 M phosphate buffered saline (PBS) for 5 min, 0.3% Triton X-100 for 5 min, 0.1 M PBS for 5 min 2 times, and 2 µg/ml proteinase K (diluted by 50 mM Tris and 2 mM calcium chloride) at 37 ℃ for 20 min. The cells were then fixed again, and treated with 0.25% acetic anhydride and 0.1 M ethanolamine for 10 min, 50% formamide/2*SSC (0.3MNaCl, 30mM Sodium Citrate) for 10 min at room temperature, and then incubated at 37 ℃ for 15 min. Pre-hybridization was performed using 100 µg/ml salmon sperm DNA and incubation at 37 ℃ for 2 hours. Finally, the samples were incubated with 400 ng/ml of probe, in hybridization buffer, at 37 ℃ overnight. The 293mid-28 probe was modified using 6-FAM at the 5’ end, and its sequence was 5’- AGATCGCACTACTGCACTCCAGCCTGGGTGACAGAGTAAGACTTCGTCTC-3’. The HeLamid-18 probe was modified using 6-TAMRA at the 5’ end and its sequence was 5’-GATGGGGGTAAATATCCAGGCTTTCCGCTCTGCTTCCATTGACACCCAAT-3’.

2.5. Pull-down assays and quantitative real-time PCR

Pull-down assays were performed according to the protocol in Reference [[20](#_ENREF_20)]. Briefly, cells were transfected with biotinylated miDNAs (50 nM; Sangon Biotech), and harvested 72 h after transfection. The cells were incubated in lysis buffer (20 mM Tris (pH 7.5), 200 mM NaCl, 2.5 mM MgCl2, 0.05% Igepal, 60 U/mL Superase-In (Ambion), 1 mM DTT, and protease inhibitors (Roche)), on ice for 30 min after washing with PBS 2 times. M-280 streptavidin magnetic beads (Sigma) were blocked by RNase-free BSA and salmon sperm DNA (both from Sigma), which were added to the lysates and incubated at 4 ℃ for 3 hours. The beads were washed three times with ice-cold lysis buffer, three times with a low salt buffer (0.1% SDS, 1% Trition X-100, 2 mM EDTA, 20 mM Tris-HCl pH 8.0, and 150 mM NaCl), and once with a high salt buffer (0.1%SDS, 1% Trition X-100, 2 mM EDTA, 20 mM Tris-HCl pH 8.0, and 500 mM NaCl). Bound RNA was extracted using Trizol (InvitrogenTM life technology) for downstream quantitative real-time PCR. The sequence of biotin-293mid-28 was 5’- TTTTTTTTTTTTTTTGAGACGAAGTCTTACTCTGTCACCCAGGCTGGAGTGCAGTAGTGCGATCTCAGCTCACTGCAACCTCCGCCTCCCAG-3’. The sequence of 293mid-54 was 5’-CAAGGTCATGAGTTTGAGATCAGCCTGGCTAATATGGTGAAACCCCATCTCTACTAAAAATACAAAAATTAGCTGGGTGTGGTGGTGGGCACCTGTAGTCCATCTACTTGGGAGAC-3’. Quantitative real-time PCR was performed as previously described [[20](#_ENREF_20)]. Briefly, miRNA levels were measured by qRT-PCR using SYBR® Premix Ex Taq™ II (Takara Clontech). miRNAs expression levels were normalized to U6 before the pull-down assay.

2.6. Cell culture and isolation of nuclei

HEK293 and HeLa cells were grown in Dulbecco’s modified Eagle’s medium (DMEM; InvitrogenTM life technology) supplemented with 10% Fetal bovine serum (FBS), 100 units/ml penicillin, and 100 μg/ml streptomycin and incubated at 37 ℃ and 5% CO2. Nuclei were isolated according to a previously described method with minor modifications [[7](#_ENREF_7)]. In this study, cells were isolated from mouse hearts (adult males, 8–12 weeks of age), mouse brains (adult males, 8–12 weeks of age), HEK293 cells, or HeLa cells. Nuclei were extracted from tissue or cell lines using sucrose gradient ultracentrifugation. Fresh tissue or cell lines were homogenized using a wheaton douncer, in buffer A (20 mM HEPES pH 7.5, 10 mM KCl, 1.5 mM MgCl2, 1 mM EGTA, 1 mM EDTA, 1 mM DTT, 0.1 mM PMSF, and 250 mM sucrose). Cell debris was removed and intact cells were pelleted after centrifugation at 800 rpm for 10 min. Nuclei were precipitated through centrifugation at 3000 rpm for 15 min.

2.7. Sequence analysis

Genome mapping, H3K27Ac activity, SNP site, and conservation level analysis were performed in the UCSC (University of California Santa Cruz) genome browser. The length distribution and GC content were analyzed by Mega software. Base-pairing analysis was performed using the bioinformatics program RNAhybrid. All statistical analyses were performed with the SPSS 16.0 software.

2.8 Detection of the endogenous linear microDNAs

HEK293 cells were transfected with biotinylated 293mid-62 antisense sequence 5’-TTGAGCATTTACCATGTTCTGGGCAGTGTGGAAGGCCTTGGGTGTATAATGTGAGCATGTGCCTGACCTGTGCTGATGGAGGAGACAAATA-3’, which was 20 nt shorter at the 5’-end than 293mid-62. The pull-down assay, based on interactions between biotin and streptavidin (described in the method—Pull-down assay and quantitative real-time PCR), was performed to purify 293mid-62. The complementary strand was synthesized and amplified by a PCR reaction with the forward primer 5’-CTTTGATCAGCATTATTTATTTTG-3’ and the reverse primer 5’-TATTTGTCTCCTCCATCAGCACAGG-3’. The amplified product was cloned into the pLB Vector (TIANGEN biotech) and sequenced. HeLamid-1 was analyzed via similar experiments with 293mid-62. The biotinylated HeLamid-1 antisense sequence was 5’-TTTTGTGGGCAACGTGACTCACTATGCCCAGGTCTGGCTCAACATCTCGGCGGAG-3’. The forward primer was 5’-CAGGCCAACGAGACTTTTGCTTTTG-3’ and the reverse primer was 5’-CTCCGCCGAGATGTTGAGCCAGACC-3’.

2.9 Transfection and detection of exogenous SSLmicroDNAs and reverse strands

The 293mid-28, HeLamid-18, and their reverse strands were synthesized by the Sangon Biotech company. The 293mid-28 and its reverse strands were modified using 6-FAM at the 5’ end. HeLamid-18 and its reverse strands were modified using 6-TAMRA at the 5’ end. The sequence of 293mid-28 was 5’-TTTTTTTTTTTTTTTGAGACGAAGTCTTACTCTGTCACCCAGGCTGGAGTGCAGTAGTGCGATCTCAGCTCACTGCAACCTCCGCCTCCCAG-3’, and its reverse strand sequence was 5’-CTGGGAGGCGGAGGTTGCAGTGAGCTGAGATCGCACTACTGCACTCCAGCCTGGGTGACAGAGTAAGACTTCGTCTCAAAAAAAAAAAAAAA-3’. The sequence of HeLamid-18 was 5’-CACCCAGCAGCAACAGGGGTCCCCCACCCCATTGGGTGTCAATGGAAGCAGAGCGGAAAGCCTGGATATTTACCCCCATCTAGAAGTAACAAG- 3’, and its reverse strand sequence was 5’-CTTGTTACTTCTAGATGGGGGTAAATATCCAGGCTTTCCGCTCTGCTTCCATTGACACCCAATGGGGTGGGGGACCCCTGTTGCTGCTGGGTG-3’. HEK293 and HeLa cells were grown on glass coverslips coated with poly-L-lysine (Sigma Aldrich). The transfection was performed using the transfection reagent Fugene (Promega), according to the manufacturer’s instructions. Cells were fixed with cold methanol at -20 ℃ for 5–10 min, and stained with DAPI. They were analyzed using a laser confocal fluorescence microscope.

3. Results

3.1. Small, single-stranded linear extrachromosomal DNAs (named SSLmicroDNAs) were identified in cell nucleus.

We purified single-stranded linear DNA using the single-stranded DNA binding protein RecAf. The DNA was extracted from the nuclei of adult mouse hearts, brains, HEK293 cells, and HeLa cells [[30](#_ENREF_30),[31](#_ENREF_31)] (Figure 1a). The purified single-stranded linear DNAs were further identified using atomic force microscopy (AFM; Figure 2a). Smear-like band distributions with a broad range were observed in agarose gel electrophoresis analysis (Figure 2b).


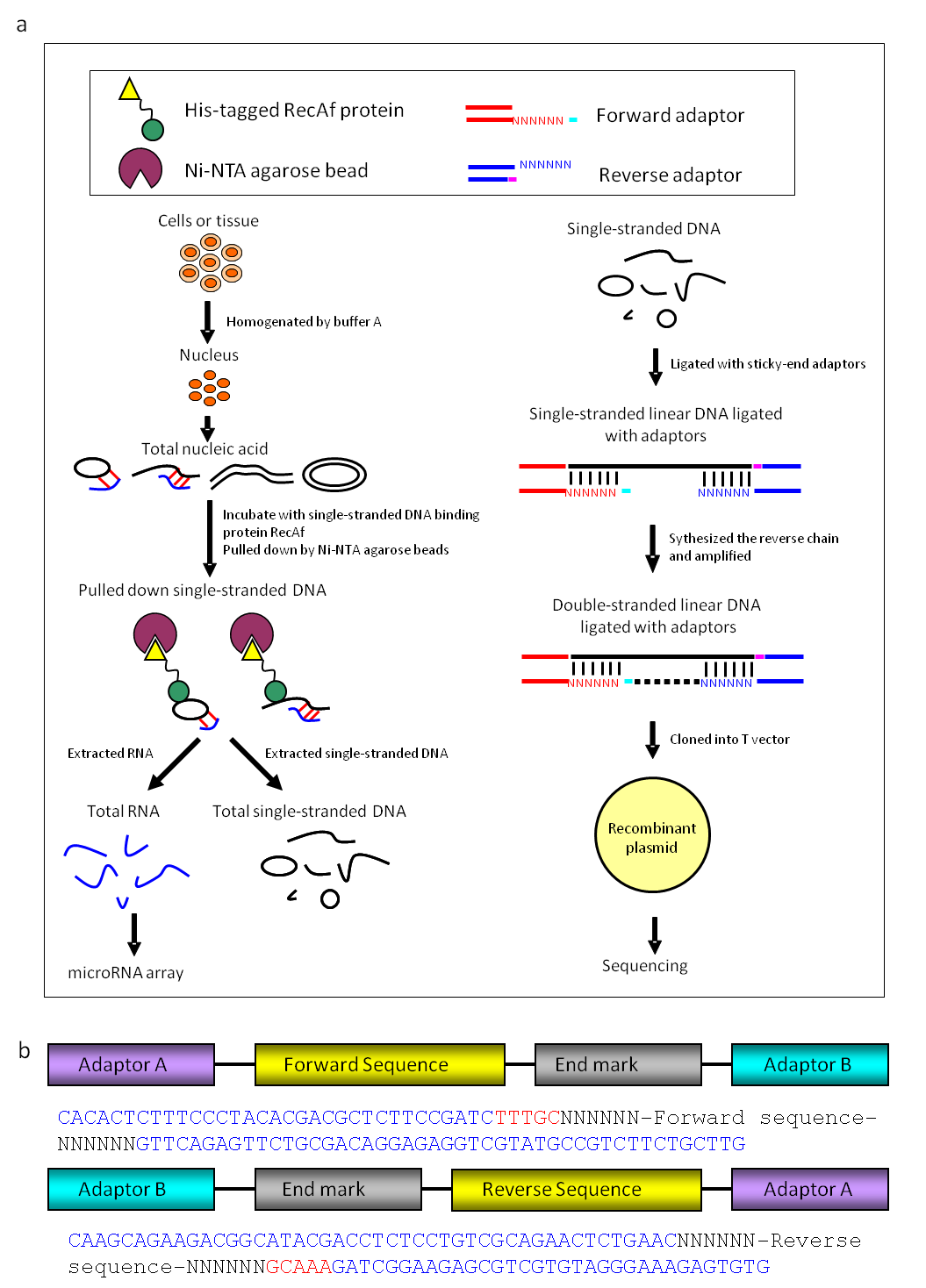


**Figure 1.** Preparation of extrachromosomal single-stranded linear microDNAs and microRNAs. a, The single-stranded linear DNAs were pulled down by the RecA_f_ protein. RNAs and single-stranded linear DNAs were isolated through pulldown system based on RecA_f_ protein. microRNA array analysis was performed with the RNA samples. Following ligated with two adaptors, the single-stranded linear DNAs libraries were constructed and sequenced. b, The composition of adaptor A and adaptor B. Adaptors composed of end markers and random bases. N represents a random base. TTTGC and GCAAA were end markers in adaptor A or B.


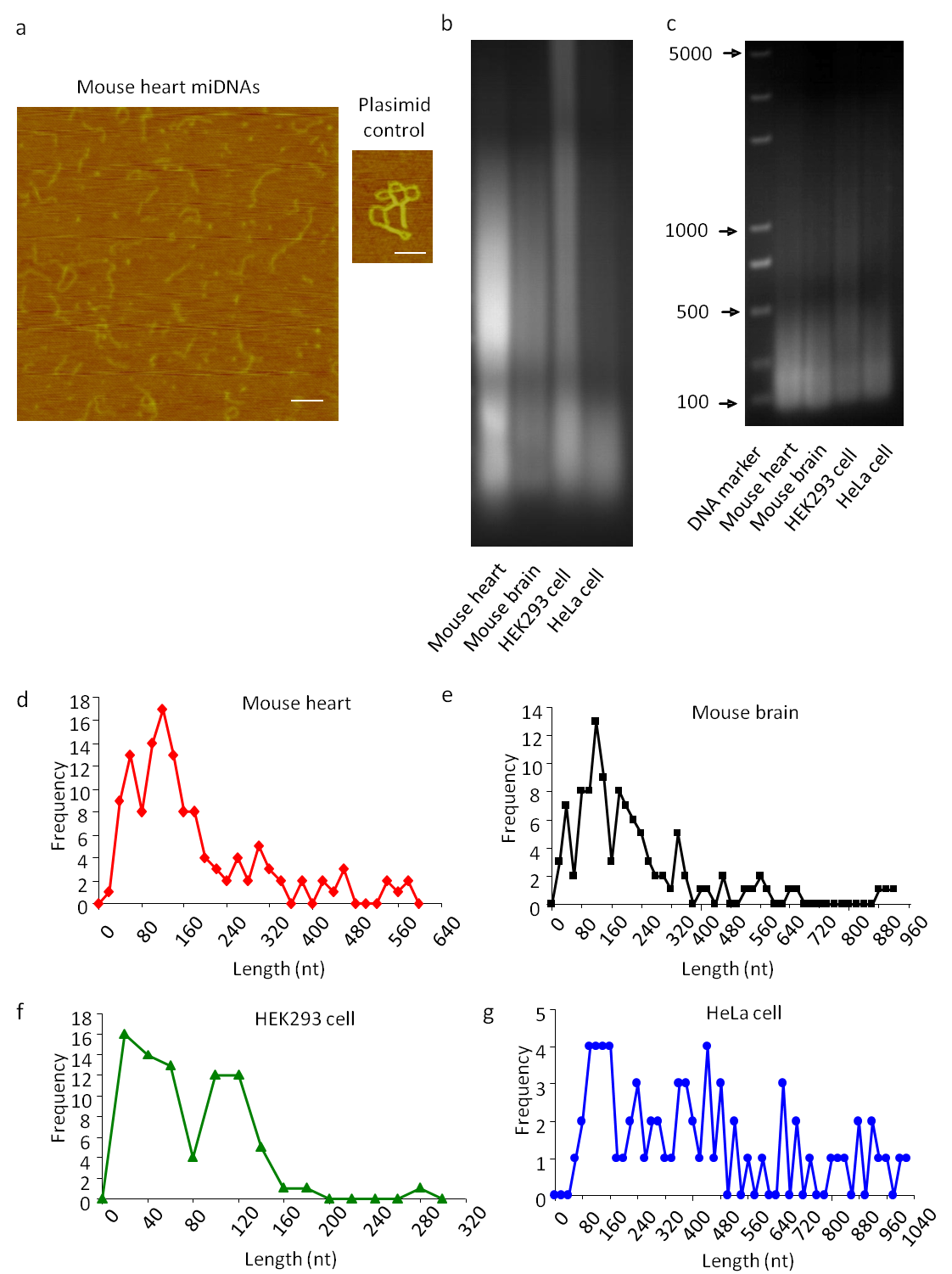


**Figure 2.** Identification of single-stranded linear extrachromosomal microDNAs. a，Analysis of mouse heart nucleic single-stranded extrachromosomal DNA by atomic force microscopy. A 2982 bp double-stranded plasmid was used as a control. Bar=200 nm. b, Analysis of single-stranded extrachromosomal DNA by agarose electrophoresis. c. Library amplification of single-stranded extrachromosomal DNA from MHSSLmicroDNA, MBSSLmicroDNA, HeLaSSLmicroDNA and 293SSLmicroDNA, respectively. d-g, Analysis of the length distribution of SSLmicroDNAs from mouse hearts (d), mouse brain (e), HEK293 cells (f) and HeLa cells (g), respectively.

To characterize linear DNAs, four libraries of single-stranded linear DNAs from mouse hearts, mouse brains, HEK293 cells, and HeLa cells were constructed. The library construction method consisted of ligating single-stranded DNA-specific adaptors, and synthesizing complementary chains (Figures 1,2c and Supplementary Figure S2). DNA sequencing analysis identified 128 single linear DNA sequences from mouse hearts (Supplementary data set 1), 107 from mouse brains (Supplementary data set 2), 78 from HEK293 cells (Supplementary data set 3), and 70 from HeLa cells (Supplementary data set 4). These data showed that mouse heart-derived linear DNA had a 16–579 nt length distribution with a peak at approximately 130 nt (Figure 2d); 50–400 nt length for mouse brain-derived linear DNA (Figure 2e); and 20–290 nt length for those derived from HEK293 cells, of which >90% were distributed between 40–150 nt (Figure 2f). However, compared to normal tissue, the length distribution of linear DNAs from HeLa cells was irregular, without a defined length peak (Figure 2d–g). Since linear DNAs have molecular weights ranging from tens to thousands, with peaks at the hundreds mark, we named them single-stranded linear microDNAs (SSLmicroDNAs). SSLmicroDNAs derived from mouse hearts, mouse brains, HEK293 cells, and HeLa cells were named MHSSLmid, MBSSLmid, 293SSLmid, and HeLaSSLmid, respectively.

3.2. SSLmicroDNAs exhibited a series of unique feature and their origins hypothesis was put forward.

To understand the nature of SSLmicroDNAs, we performed detailed characterization using statistical and bioinformatic methods. We conducted a comparative analysis of all 383 SSLmicroDNA sequences in the UCSC genome browser (Figure S3). The results showed >90% of these sequences were located in non-coding regions, including intergenic and intragenic regions (Figure 3a). Analysis of microDNA loci and their neighboring regions showed that the 42.5% GC content of HeLaSSLmids was slightly higher than the 37.5% GC content of their 500 bp up or downstream neighboring regions (Figure 3b). MHSSLmids had a similar GC content to HeLaSSLmids, while MBSSLmids and 293SSLmids did not (Figure 3b). Remarkably, AT, TT, or AA dinucleotides appeared periodically, every 10 bp along the microDNA sequence, and were identified in approximately 76.64% of 293SSLmids, 64.84% of MHSSLmids, 57.01% of MBSSLmids, and 38.57% of HeLaSSLmids (Figure 3c). The periodicity of microDNAs isolated from normal tissues was much greater than those from tumor cells (Figure 3c). A previous study revealed that AT, TT, or AA dinucleotide periodicity usually appeared in sequences assembled into nucleosomes [[32](#_ENREF_32)]. Therefore, we hypothesized that SSLmicroDNAs may originate from nucleosomes.


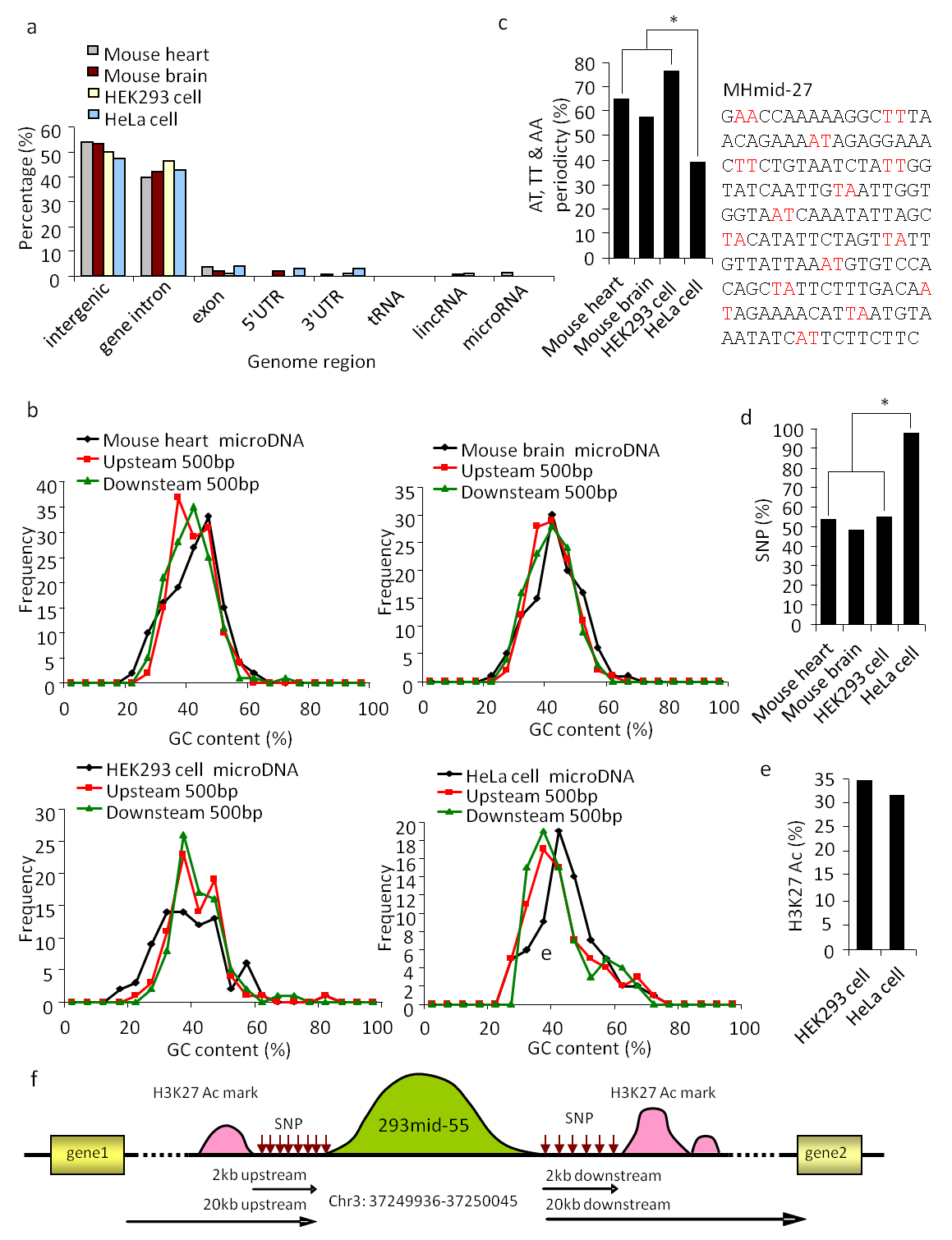


**Figure 3.** Properties of SSLmicroDNA sequences and their genomic loci. a, Genome mapping of SSLmicroDNAs from mouse hearts, mouse brains, HEK293 cells and HeLa cells. b, GC content of SSLmicroDNAs and their 500 bp-upstream and 500 bp-downstream regions. c, Analysis of AT, TT or AA periodicity of SSLmicroDNA sequences from mouse hearts, mouse brains, HEK293 cells and HeLa cells. The MHmid-27 sequence periodically separated by AT, TT or AA is presented. **p<0.05*. d, Analysis of single nucleotide polymorphism sites (SNPs). The data are the percentages of microDNA loci with SNPs within 2kb upstream or downstream. **p<0.05.* e, Analysis of H3K27 Ac marks within 2 kb-upstream or -downstream regions of SSLmicroDNAs genomic loci. The data are the percentages of microDNA loci that have H3K27 Ac marks within the indicated regions. f, A glance at the scenery near 293mid-55 in the genome.

Single nucleotide polymorphisms (SNPs) refer to changes in single nucleotides of DNA, which lead to genome diversity and instability [[33](#_ENREF_33)]. Our results showed that ~50% of microDNAs in mouse heart, mouse brain, and HEK293 cells, and >90% in HeLa cells were located in SNP-rich regions—2 kb upstream or downstream (Figure 3d,f). H3K27Ac chromatin marks, usually appear at the site of active regulatory elements, such as gene enhancers and promoters [[34](#_ENREF_34)]. Approximately 30% of SSLmicroDNAs from HEK293 and HeLa cells were located near high-H3K27Ac-activity regions (Figure 3e,f), which led us to hypothesize that H3K27Ac-marked regions could be a source for SSLmicroDNA production. Taken together, these data indicate that SSLmicroDNAs are likely generated from regions with strong H3K27Ac marks, and frequent single nucleotide polymorphisms.

To further confirm the type of purified extrachromosomal DNA; single-stranded or linear, we designed a pull-down system based on the interaction between biotin and streptavidin (Supplementary Figure S1). The results showed that 293mid-62 was pulled down by a 293mid-62 probe modified with a biotin tag at the 5’-end, and HeLamid-1 was pulled down by its respective probe (Figure 4a).


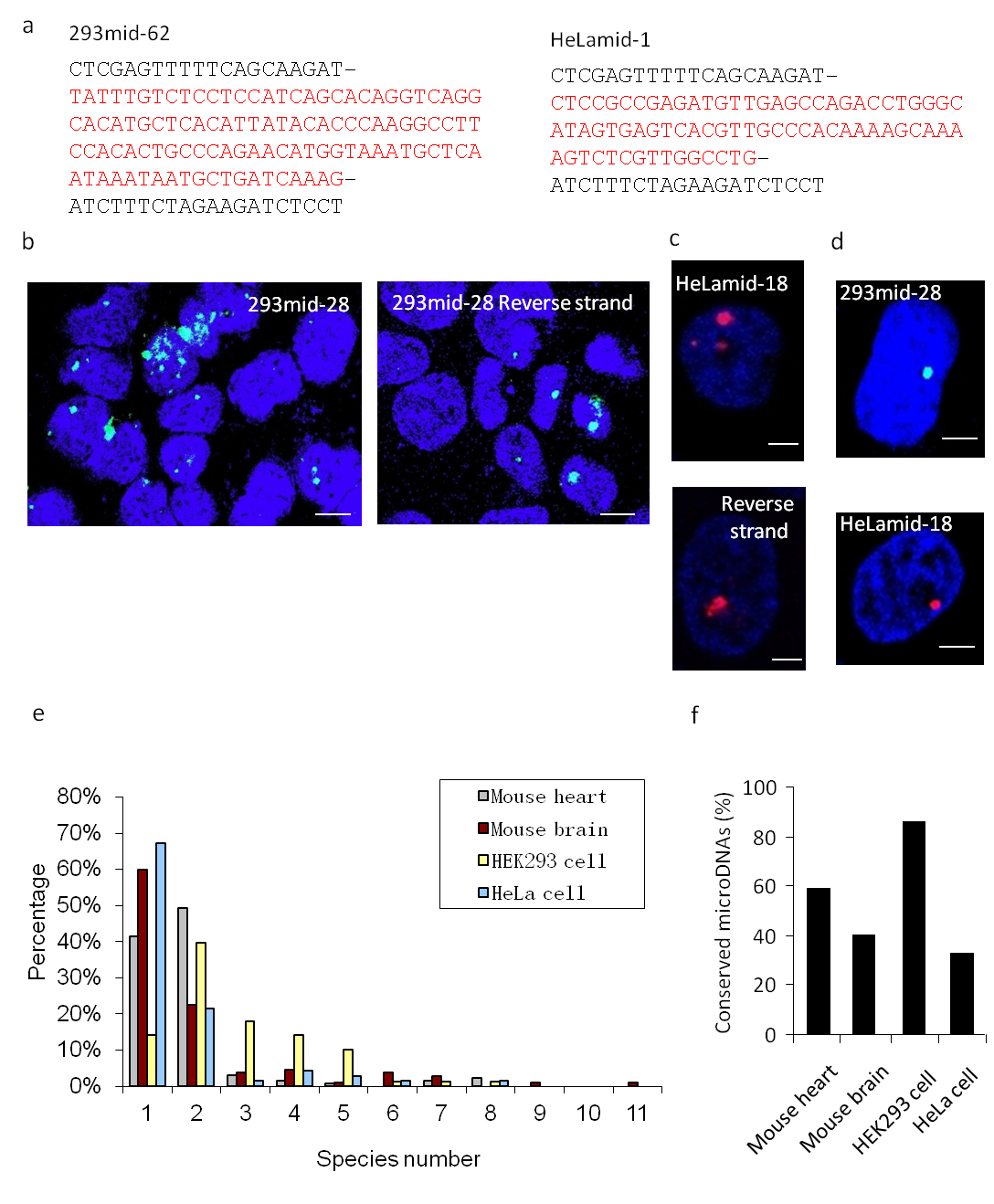


**Figure 4.** Determination of microDNA subcellular localization and conservation level. a, The sequencing data of 293mid-62 and HeLamid-1 in the detection of endogenous linear microDNAs. The 293mid-62 probe with a biotin tag at the 5’-end was transfected into HEK293 cells, and HeLamid-1 probe with a biotin tag at the 5’-end was transfected into HeLa cells. Then the pulldown system based on interaction between biotin and streptavidin was performed and the 293mid-62 and HeLamid-1 were detected by sequencing. b-c, Detection of SSLmicroDNA subcellular localization. HEK293 cells were transfected with 293mid-28 or its reverse strands labeled with a FAM at the 5’-end (b), and HeLa cells were transfected with HeLamid-18 or its reverse strands labeled with a TAMRA at the 5’-end (c). The nuclei were stained by DAPI (the dark blue area). The light blue dots represented 293mid-28 or its reverse strands. The red dots represented HeLamid-18 or its reverse strands. The Bar=10 μm. d, Fluorescence *in situ* hybridization (FISH) analysis of SSLmicroDNA localization in cell nucleus. 293mid-28 was detected by its probes labeled with FAM at the 5’-end in HEK293 cell nucleus. HeLamid-18 was detected by its probes labeled with TAMRA at the 5’-end in HeLa cell nuclei. The nuclei were stained by DAPI (the dark blue area). The light blue dots represented 293mid-28. The red dots represented HeLamid-18. Bar=20 μm. e, Analysis of the conservation level of SSLmicroDNA sequences. Species number represents the number of species in which SSLmicroDNAs were conserved. f, Summary of the conservation level of SSLmicroDNAs. The SSLmicroDNAs that were conserved in more than one species were counted.

3.3. Cell nulei- located SSLmicroDNAs owned a high conservation level.

We then studied the subcellular localization of microDNAs, 293mid-28 and HeLamid-18 were chosen randomly. Twenty-four hours post transfection, both 293mid-28 and its reverse strand were observed in the nuclei of HEK293 cells (Figure 4b). HeLamid-18 and its reverse strand were transfected into HeLa cells, and were also found in the nucleus (Figure 4c). These results were further confirmed by fluorescence in situ hybridization (FISH), showing endogenous 293mid-28 and HeLamid-18 in the nuclei (Figure 4d).

After analyzing the conservation levels of SSLmicroDNAs, we found that >80% of HEK293SSLmicroDNAs, ~60% of MHSSLmicroDNAs, and ~40% of MBSSLmicroDNAs were conserved between two or more species, whereas only 32.86% of HeLaSSLmicroDNAs shared this feature (Figure 4e,f). These results indicated that SSLmicroDNAs in normal tissues or cells are slightly more conserved compared to their tumor-derived counterparts.

3.4. SSLmicroDNAs interacted with microRNAs in cell nucleus.

Many miRNAs are distributed in nuclei, where they can regulate chromatin remodeling, transcriptional silencing, mRNA alternative splicing, and microRNA maturation [[23-25](#_ENREF_23),[27](#_ENREF_27)]. The abundance of SSLmicroDNAs in the nuclei of normal tissues and cells suggested a potential participation in microRNA regulatory pathways. Therefore, we studied the interaction between microDNAs and microRNAs. MicroRNAs were extracted from the nuclear pull-down product via the single-stranded DNA binding protein RecAf and analyzed using microRNA arrays. MicroRNA arrays identified 421 mouse heart (Table S1), 285 mouse brain (Table S2), and 1101 HEK293 microRNAs (Table S3). We analyzed all of the microRNA and SSLmicroDNA sequences using the bioinformatics program RNAhybrid. The results showed that microDNAs pair well to at least one microRNA (Figure 5a–c, and Supplementary Figures S4–S6). Among all the microDNA–microRNA sequence alignments, we detected ~45.76% hybridization with more than 20 kcal/mol free energy in the mouse brain library, and 36.38% in the mouse heart library (Figure 5d). Furthermore, 24.41% of the hybridizations between microDNAs and microRNAs in mouse hearts, 28.97% in mouse brains, and 27.27% in HEK293 cells exhibited more than 6 bp-continuous base pairing at microRNA seed regions. At any given location, 10 bp-continuous base pairing appeared in 56.69% of the hybridizations in the mouse heart, 58.88% in mouse brains, and 66.23% in HEK293 cells (Figure 5e). The microRNAs seed region is a critical recognition and functional site for targeting to mRNAs [[35](#_ENREF_35)]. Therefore, we hypothesized that this was also a site for microDNAs interaction with microRNAs in the cell’s nucleus. To validate this hypothesis, we performed pull-down experiments, and showed that mir-1273g-3p was pulled down by the 293mid-28, and mir-5096 was pulled down by the 293mid-54, which was both interrupted by the mutation in the predicted target region of microDNA (Figure 5f). Taken together, these data indicate that SSLmicroDNAs likely interact with microRNAs in nuclei.


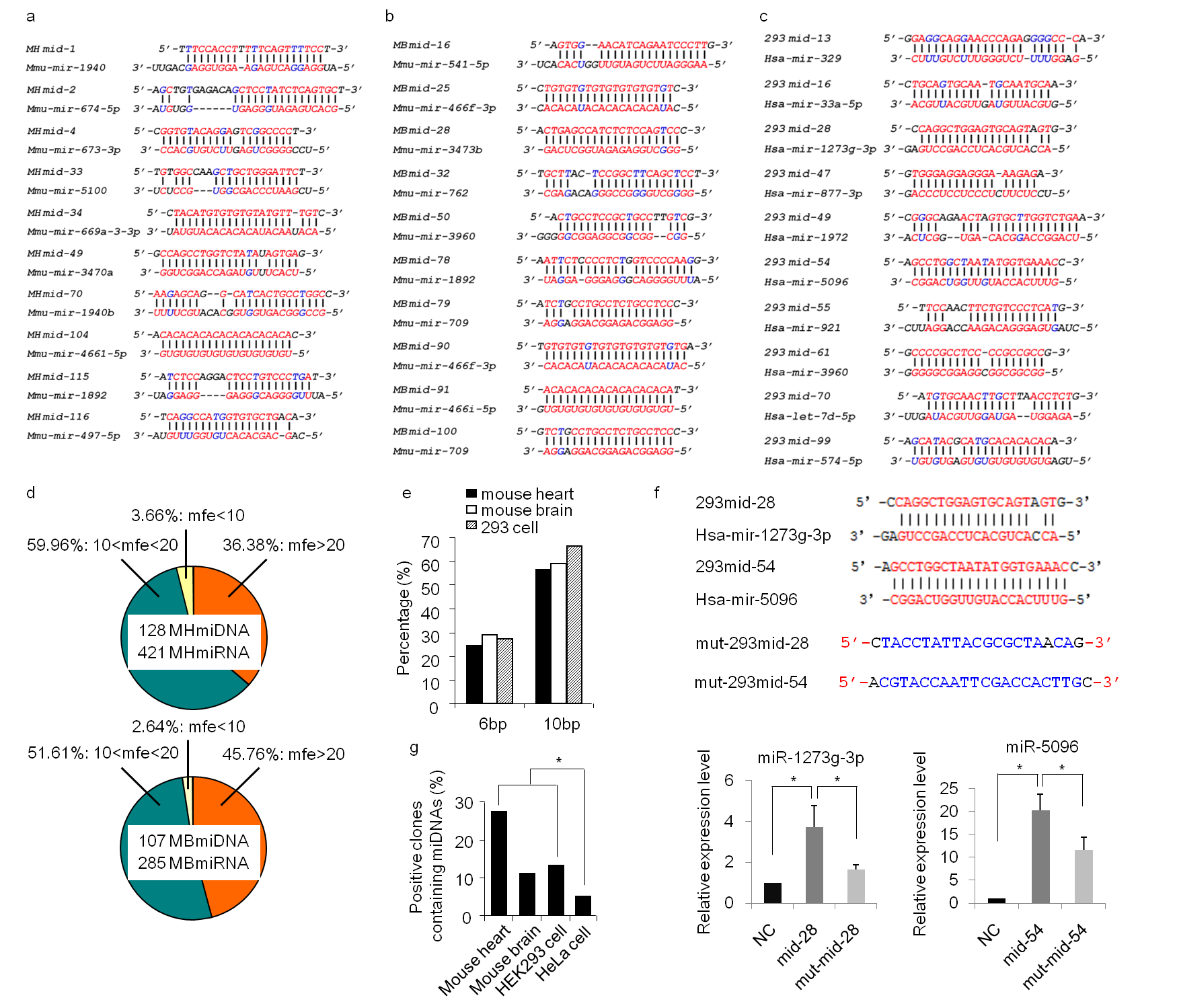


**Figure 5.** Interaction between microDNAs and microRNAs. a-c, Sequence alignments between SSLmicroDNAs and microRNAs in HEK293 cells (a), mouse hearts (b) and mouse brains (c) were analyzed using the bioinformatics program RNAhybrid. d, Analysis of the free energy (mfe) in microDNA-microRNA alignments. The unit of energy was –kcal/mol. e, Analysis of the microDNA-microRNA sequence alignment. 6 bp represented 6 bp- or more than 6 bp-continuous pairing in the seed region of microRNAs. 10 bp represented 10 bp- or more than 10 bp-continuous pairing in microRNAs. f, Detection of the interaction between microDNAs and microRNAs in cell nucleus. The upper panel showed the sequence alignment we predicted between 293mid-28 and hsa-mir-1273g-3p, as well as 293mid-54 and hsa-mir-5096. The middle panel showed the mutant sequence region in 293mid-28 and 293mid-54. HEK293 cells were transfected with biotinylated 293mid-28 or negative control RNA (NC) or mutant 293mid-28 and then performed with Pulldown system based on interaction between biotin and streptavidin. The levels of miR-1273g-3p were detected by qRT-PCR in the Pulldown product. n=3, *p<0.05 vs NC. The combination of 293mid-54 and miR-5096 was analyzed similarly to that of 293mid-28. g, Analysis of SSLmicroDNA yield. The rates of SSLmicroDNA-positive clones relative to the total number of clones during purification were counted. **p<0.05.*

4. Discussion

Extrachromosomal DNAs exist in diversiform and are ubiquitously expressed in vivo, however, little is known about their function. Our present work identified a new form of extrachromosomal linear DNAs found in normal tissue and tumor cells. These DNAs were characterized by linear single strands, tens to hundreds of nucleotides length, a little higher than average GC content periodically intercepted by AT, TT, or AA dinuleotides. The single-stranded linear microDNAs were mainly localized at the non-coding regions of genome, where various SNP sites and strong H3K27Ac marks existed. SSLmicroDNAs, located in nuclei, were shown to interact with microRNAs in vivo. The difference in microDNAs content between normal tissues and tumor cells suggest that microDNAs may have a function in cellular defence against tumorgenesis. Briefly, our results revealed a new form of extrachromosomal DNAs, which could interact with microRNAs in the cell nucleus.

Circular microDNAs have been identified in normal tissues, and exhibit unique sequence features [[7](#_ENREF_7)]. Compared to circular microDNAs, linear microDNAs own a similar length distribution and GC content; both forms shared AT, TT, or AA dinucleotide periodicity; circular microDNAs originate predominantly from functional regions such as exons, 5’ UTRs, 3’ UTRs, and CpG regions [[7](#_ENREF_7)], whereas linear microDNAs are mainly produced from non-coding regions, such as intergenic or intragenic regions of genome.

Attention has been focused on extrachromosomal DNAs’ general mechanisms of action, with no clear understanding as of yet. Viral DNA likely fails to integrate into host genomes and remains outside host chromosomes during viral infection, of which a typical example is human immunodeficiency virus (HIV) [[36](#_ENREF_36)]. Additionally, DNA of the adeno-associated virus (AAV) exists mainly as circular episomes in human tonsil-adenoid, spleen, and lung tissues after infection [[37](#_ENREF_37)]. Non-integrated Moloney murine leukemia virus (M-MuLV) DNA has been identified in a human rhabdomyosarcoma cell line (TE671 subline) [[38](#_ENREF_38)]. Therefore, a bold hypothesis about the origin of linear microDNAs is that they could evolve from infectious organisms.

Cell defence mainly relies on the immune system [[39](#_ENREF_39)]. DNA-protein crosslinking and non-coding RNAs have been shown to function directly in regulating cell defence [[40](#_ENREF_40),[41](#_ENREF_41)]. Interestingly, HeLamicroDNAs demonstrated an irregular length distribution (Figure 2d–g) and lower yield during the extraction of SSLmicroDNAs (Figure 5g, and Table 1) from HeLa cells compared to normal tissues. This indicated that microDNAs may participate in maintaining the physiological environment against tumorigenesis.

**Table 1. Summary of single-stranded microDNA library construction**

|  | **The number of clones** | **The number of sequenced clones** | **The number of clones containing sequences** | **The number of clones containing SDLmicroDNAs** | **Rate** |
| --- | --- | --- | --- | --- | --- |
| **Mouse hearts** | 3140 | 470 | 165 | 128 | 27.23% |
| **Mouse brains** | 3692 | 966 | 274 | 107 | 11.08% |
| **HEK293 cells** | 4900 | 702 | 642 | 78 | 12.15% |
| **HeLa cells** | 4700 | 2020 | 331 | 70 | 3.47% |

In summary, we identified a new form of extrachromosomal microDNAs and analyzed their characteristics. SSLMicroDNAs are located in the cell nucleus, and interacted with microRNAs. Our present work revealed a new kind of regulatory molecule that functions during physiological processes in cells, through targeting small non-coding RNAs.

**Supplementary Materials:** The following are available online at www.mdpi.com/xxx/s1, Figure S1: title, Table S1: title, Video S1: title.

**Author Contributions:** conceptualization, P.-F.L., J.-X.W. and T.S.; methodology, T.S., J.-X.W., K.W., C.-Y.L. and Y.W.; software, T.S.; validation, P.-F.L., J.-X.W. and T.S.; formal analysis, P.-F.L., J.-X.W. and T.S.; investigation, T.S., K.W. and C.-Y.L.; resources, P.-F.L., J.-X.W., T.S., K.W., C.-Y.L. and Y.W.; data curation, P.-F.L. and T.S.; writing—original draft preparation, T.S.; writing—review and editing, P.-F.L.; visualization, P.-F.L. and T.S.; supervision, P.-F.L., J.-X.W.; project administration, P.-F.L., J.-X.W. and T.S.; funding acquisition, P.-F.L. and T.S.

**Funding:** This research was funded by the National Natural Science Foundation of China (81800268, 31430041), supported by Applied Basic Research Program of Shanxi Province (201801D221268), Scientific and Technologial Innovation Programs of Higher Education Institutions in Shanxi (STIP)(201802060), and China Postdoctoral Science Foundation (2016M592134).

**Conflicts of Interest:** The authors declare no conflict of interest.

References

[1] Kuttler, F.; Mai, S. In *Formation of non-random extrachromosomal elements during development, differentiation and oncogenesis*, Seminars in cancer biology, 2007; Elsevier: pp 56-64.

[2] Handa, H. Linear plasmids in plant mitochondria: Peaceful coexistences or malicious invasions? *Mitochondrion* **2008**, *8*, 15-25.

[3] Sinclair, D.A.; Guarente, L. Extrachromosomal rdna circles--a cause of aging in yeast. *Cell* **1997**, *91*, 1033-1042.

[4] Huber, D.; Rustchenko, E. Large circular and linear rdna plasmids in candida albicans. *Yeast (Chichester, England)* **2001**, *18*, 261-272.

[5] Casjens, S.; Palmer, N.; van Vugt, R.; Huang, W.M.; Stevenson, B.; Rosa, P.; Lathigra, R.; Sutton, G.; Peterson, J.; Dodson, R.J.*, et al.* A bacterial genome in flux: The twelve linear and nine circular extrachromosomal dnas in an infectious isolate of the lyme disease spirochete borrelia burgdorferi. *Molecular microbiology* **2000**, *35*, 490-516.

[6] Shadel, G.S.; Clayton, D.A. Mitochondrial DNA maintenance in vertebrates. *Annual review of biochemistry* **1997**, *66*, 409-435.

[7] Shibata, Y.; Kumar, P.; Layer, R.; Willcox, S.; Gagan, J.R.; Griffith, J.D.; Dutta, A. Extrachromosomal microdnas and chromosomal microdeletions in normal tissues. *Science (New York, N.Y.)* **2012**, *336*, 82-86.

[8] Cox, D.; Yuncken, C.; Spriggs, A.I. Minute chromatin bodies in malignant tumours of childhood. *Lancet (London, England)* **1965**, *1*, 55-58.

[9] Hahn, P.J. Molecular biology of double-minute chromosomes. *BioEssays : news and reviews in molecular, cellular and developmental biology* **1993**, *15*, 477-484.

[10] Shimizu, N.; Ochi, T.; Itonaga, K. Replication timing of amplified genetic regions relates to intranuclear localization but not to genetic activity or g/r band. *Experimental cell research* **2001**, *268*, 201-210.

[11] Turner, K.M.; Deshpande, V.; Beyter, D.; Koga, T.; Rusert, J.; Lee, C.; Li, B.; Arden, K.; Ren, B.; Nathanson, D.A.*, et al.* Extrachromosomal oncogene amplification drives tumour evolution and genetic heterogeneity. *Nature* **2017**, *543*, 122-125.

[12] Starling, S. Cancer genomics: Ecdetect hunts extrachromosomal DNA. *Nature reviews. Genetics* **2017**, *18*, 212.

[13] Dillon, L.W.; Kumar, P.; Shibata, Y.; Wang, Y.H.; Willcox, S.; Griffith, J.D.; Pommier, Y.; Takeda, S.; Dutta, A. Production of extrachromosomal microdnas is linked to mismatch repair pathways and transcriptional activity. *Cell reports* **2015**, *11*, 1749-1759.

[14] Cara, A.; Reitz, M.S., Jr. New insight on the role of extrachromosomal retroviral DNA. *Leukemia* **1997**, *11*, 1395-1399.

[15] Matuszewski, B.; Ciechomski, K.; Kloc, M. Extrachromosomal rdna and polarity of pro-oocytes during ovary development in creophilus maxillosus (coleoptera, staphylinidae). *Folia histochemica et cytobiologica* **1999**, *37*, 179-190.

[16] McDermott, P.; Connolly, V.; Kavanagh, T.A. The mitochondrial genome of a cytoplasmic male sterile line of perennial ryegrass (lolium perenne l.) contains an integrated linear plasmid-like element. *TAG. Theoretical and applied genetics. Theoretische und angewandte Genetik* **2008**, *117*, 459-470.

[17] Rose, K.; Fetzner, S. Identification of linear plasmid pam1 in the flavonoid degrading strain actinoplanes missouriensis(t) (dsm 43046). *Plasmid* **2006**, *55*, 249-254.

[18] Palmer, N.; Fraser, C.; Casjens, S. Distribution of twelve linear extrachromosomal dnas in natural isolates of lyme disease spirochetes. *Journal of bacteriology* **2000**, *182*, 2476-2480.

[19] Sun, T.; Dong, Y.H.; Du, W.; Shi, C.Y.; Wang, K.; Tariq, M.A.; Wang, J.X.; Li, P.F. The role of micrornas in myocardial infarction: From molecular mechanism to clinical application. *International journal of molecular sciences* **2017**, *18*.

[20] Wang, K.; Sun, T.; Li, N.; Wang, Y.; Wang, J.X.; Zhou, L.Y.; Long, B.; Liu, C.Y.; Liu, F.; Li, P.F. Mdrl lncrna regulates the processing of mir-484 primary transcript by targeting mir-361. *PLoS genetics* **2014**, *10*, e1004467.

[21] Thum, T.; Gross, C.; Fiedler, J.; Fischer, T.; Kissler, S.; Bussen, M.; Galuppo, P.; Just, S.; Rottbauer, W.; Frantz, S.*, et al.* Microrna-21 contributes to myocardial disease by stimulating map kinase signalling in fibroblasts. *Nature* **2008**, *456*, 980-984.

[22] Bartel, D.P. Micrornas: Genomics, biogenesis, mechanism, and function. *Cell* **2004**, *116*, 281-297.

[23] Zhang, X.; Li, H.; Burnett, J.C.; Rossi, J.J. The role of antisense long noncoding rna in small rna-triggered gene activation. *RNA (New York, N.Y.)* **2014**, *20*, 1916-1928.

[24] Kim, D.H.; Saetrom, P.; Snove, O., Jr.; Rossi, J.J. Microrna-directed transcriptional gene silencing in mammalian cells. *Proceedings of the National Academy of Sciences of the United States of America* **2008**, *105*, 16230-16235.

[25] Kalsotra, A.; Wang, K.; Li, P.F.; Cooper, T.A. Micrornas coordinate an alternative splicing network during mouse postnatal heart development. *Genes & development* **2010**, *24*, 653-658.

[26] Allo, M.; Buggiano, V.; Fededa, J.P.; Petrillo, E.; Schor, I.; de la Mata, M.; Agirre, E.; Plass, M.; Eyras, E.; Elela, S.A.*, et al.* Control of alternative splicing through sirna-mediated transcriptional gene silencing. *Nature structural & molecular biology* **2009**, *16*, 717-724.

[27] Zisoulis, D.G.; Kai, Z.S.; Chang, R.K.; Pasquinelli, A.E. Autoregulation of microrna biogenesis by let-7 and argonaute. *Nature* **2012**, *486*, 541-544.

[28] Faas, F.G.; Rieger, B.; van Vliet, L.J.; Cherny, D.I. DNA deformations near charged surfaces: Electron and atomic force microscopy views. *Biophysical journal* **2009**, *97*, 1148-1157.

[29] Rowley, J.D.; Diaz, M.O.; Espinosa, R., 3rd; Patel, Y.D.; van Melle, E.; Ziemin, S.; Taillon-Miller, P.; Lichter, P.; Evans, G.A.; Kersey, J.H.*, et al.* Mapping chromosome band 11q23 in human acute leukemia with biotinylated probes: Identification of 11q23 translocation breakpoints with a yeast artificial chromosome. *Proceedings of the National Academy of Sciences of the United States of America* **1990**, *87*, 9358-9362.

[30] Fu, H.; Le, S.; Chen, H.; Muniyappa, K.; Yan, J. Force and atp hydrolysis dependent regulation of reca nucleoprotein filament by single-stranded DNA binding protein. *Nucleic acids research* **2013**, *41*, 924-932.

[31] Ha, T.; Kozlov, A.G.; Lohman, T.M. Single-molecule views of protein movement on single-stranded DNA. *Annual review of biophysics* **2012**, *41*, 295-319.

[32] Segal, E.; Fondufe-Mittendorf, Y.; Chen, L.; Thastrom, A.; Field, Y.; Moore, I.K.; Wang, J.P.; Widom, J. A genomic code for nucleosome positioning. *Nature* **2006**, *442*, 772-778.

[33] Gaasenbeek, M.; Howarth, K.; Rowan, A.J.; Gorman, P.A.; Jones, A.; Chaplin, T.; Liu, Y.; Bicknell, D.; Davison, E.J.; Fiegler, H.*, et al.* Combined array-comparative genomic hybridization and single-nucleotide polymorphism-loss of heterozygosity analysis reveals complex changes and multiple forms of chromosomal instability in colorectal cancers. *Cancer research* **2006**, *66*, 3471-3479.

[34] Creyghton, M.P.; Cheng, A.W.; Welstead, G.G.; Kooistra, T.; Carey, B.W.; Steine, E.J.; Hanna, J.; Lodato, M.A.; Frampton, G.M.; Sharp, P.A.*, et al.* Histone h3k27ac separates active from poised enhancers and predicts developmental state. *Proceedings of the National Academy of Sciences of the United States of America* **2010**, *107*, 21931-21936.

[35] Johnnidis, J.B.; Harris, M.H.; Wheeler, R.T.; Stehling-Sun, S.; Lam, M.H.; Kirak, O.; Brummelkamp, T.R.; Fleming, M.D.; Camargo, F.D. Regulation of progenitor cell proliferation and granulocyte function by microrna-223. *Nature* **2008**, *451*, 1125-1129.

[36] Sloan, R.D.; Wainberg, M.A. The role of unintegrated DNA in hiv infection. *Retrovirology* **2011**, *8*, 52.

[37] Schnepp, B.C.; Jensen, R.L.; Chen, C.L.; Johnson, P.R.; Clark, K.R. Characterization of adeno-associated virus genomes isolated from human tissues. *Journal of virology* **2005**, *79*, 14793-14803.

[38] Schneider, W.M.; Wu, D.T.; Amin, V.; Aiyer, S.; Roth, M.J. Mulv in mutants responsive to hdac inhibitors enhance transcription from unintegrated retroviral DNA. *Virology* **2012**, *426*, 188-196.

[39] Weiss, G.; Schaible, U.E. Macrophage defense mechanisms against intracellular bacteria. *Immunological reviews* **2015**, *264*, 182-203.

[40] Lecellier, C.H.; Dunoyer, P.; Arar, K.; Lehmann-Che, J.; Eyquem, S.; Himber, C.; Saib, A.; Voinnet, O. A cellular microrna mediates antiviral defense in human cells. *Science (New York, N.Y.)* **2005**, *308*, 557-560.

[41] Stingele, J.; Schwarz, M.S.; Bloemeke, N.; Wolf, P.G.; Jentsch, S. A DNA-dependent protease involved in DNA-protein crosslink repair. *Cell* **2014**, *158*, 327-338.

| 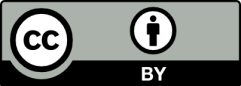 | © 2019 by the authors. Submitted for possible open access publication under the terms and conditions of the Creative Commons Attribution (CC BY) license (http://creativecommons.org/licenses/by/4.0/). |
| --- | --- |
